# Polygenic Scores of Executive Function Provide Limited Support for Genetic Confounding With Socio‐Economic Measures

**DOI:** 10.1111/gbb.70030

**Published:** 2025-07-23

**Authors:** Lucas C. Perry, Nicolas Chevalier, Michelle Luciano

**Affiliations:** ^1^ School of Philosophy, Psychology and Language Sciences University of Edinburgh Edinburgh UK

**Keywords:** executive function, genetic confounding, indirect genetic effects, maternal education, polygenic scores, prenatal smoking, socio‐economic status

## Abstract

Previous work has suggested that genetic confounding is a persistent issue in studies of environmental predictors of executive function (EF). This is largely because controlling for genetic confounding typically requires specialized samples such as twins or adoptees, which are more difficult to recruit. Polygenic scores provide a potential alternative control, scalable to smaller samples and not requiring specialized sample features. The purpose of this study was to determine if polygenic scores of EF could be used to replicate the findings of other genetic confounding studies in a less specialized sample. Confounding models showed evidence for genetic confounding in maternal education, although it was far weaker in magnitude than in other genetically informed studies. However, consistent with previous research, there were no detectable influences of indirect genetic effects on the EF polygenic score, indicating that the detected genetic confounding was likely a true genetic effect. Finally, while environmental factors other than maternal education seemed predictive of EF, confounding models showed that this was best explained by their association with maternal education. Other predictors of EF may thus be confounded environmentally, not just genetically. While polygenic scores are a promising method with a multitude of applications, in their current state they do not replicate the findings of other genetically informed studies of EF. Caution should thus be used when employing them to study genetic confounding in EF.

## Graphical abstract

While there is good reason to believe EF research has been subject to genetic confounding, the specialized samples required to test for it can be impractical for researchers. We tested one proposed alternative genetic control, polygenic scores, finding that they produced modest evidence for genetic confounding inconsistent with other literature, suggesting they should not be recommended for use as such.

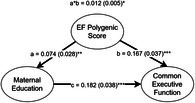

## 1 Introduction

Executive function (EF) has attracted attention as a potential broad‐spectrum intervention target for a variety of outcomes ([1]), leading to research interested in identifying early‐life determinants of the trait. Maternal education, socioeconomic status [2], prenatal smoking [3], and single parenthood [4, 5, 6] have all been studied and found to significantly predict EF outcomes. However, such research has largely relied on correlational designs that cannot assess causality. And given that this research has been attempting to examine environmental transmission between genetic relatives, it is possible that much of these findings can be explained by genetic confounding [7, 8, 9]. Genetic confounding can occur when traits in a parent and traits in their offspring are influenced by the same genes present in both. The resulting correlation between these traits (or associated outcomes) can give the appearance of environmental causation, but the true relationship is genetic. This is particularly relevant to EF because many of the environments that have been associated with offspring EF outcomes are themselves likely influenced by parental EF. For example, EF is believed to aid academic achievement [10, 11, 12], meaning that parents with better EF are more likely to attain more advanced educational qualifications. So, while it may be that the environments supplied by more educated parents are more conducive to EF development, it is also possible that this association is simply an artifact of genetic transmission of EF from parent to child.

An early clue indicating this possibility has come from twin studies, which have suggested that variance in EF is largely due to genetics, and to a lesser extent non‐shared environmental influences [13, 14, 15, 16, 17, 18]. On the one hand, twin studies estimating such influences cannot identify causal associations with particular genes or particular environments, and supporting evidence from other designs such as adoption or family studies is lacking in the literature. However, if twin study estimates of genetic, shared environmental, or non‐shared environmental influences on a trait are very low, this might suggest that predictors falling into that category are unlikely to be major determinants of that trait [19]. Therefore, the common finding of low estimates for shared environmental influence on EF runs contrary to causal interpretations of the observed correlations between EF and the family environment. There are admittedly several limitations of twin studies that may cause them to underestimate shared environment—the classical twin design can underestimate variance due to shared environment as a consequence of unmodeled non‐additive genetic effects [20], which has received some limited support from a combined twin and adoption study of EF (which found non‐significant but non‐zero estimates for the influence of shared environment on EF, [21]). Furthermore, homogenous samples will miss variation due to environments not present in their sample, limiting generalizability. But even if the finding of low shared environmental influence is not strictly true, the finding that EF is a heritable trait is sufficient to suspect that genetic confounding may be operable in its associations [7, 8, 9].

Indeed, genetically informed studies of EF have generally supported the claim that genetic confounding is the better explanation for these environmental associations. Discordant sibling designs [22, 23], adoption designs [24], and bivariate genome‐wide complex trait analysis (GCTA) using molecular genetic data [25] have all largely supported this conclusion. However, despite the pervasiveness of potentially genetically confounded designs in EF literature, there are few studies that examine this risk, likely due to the relatively specialized nature of methods used to control for it. Twin and adoption designs rely on family structures uncommon in the general population, and discordant sibling exposures may be rare or even impossible depending on the variable of interest. Even bivariate GCTA, while in principle easily applied to general population samples, is impractical for most studies, as it typically requires sample sizes exceeding several thousand to achieve sufficient statistical power [26].

For this reason, some works concerned with encouraging researchers to control for genetic confounding have suggested polygenic scores as an alternative, more accessible method [7, 8, 9]. Polygenic scores aggregate the effect of small, common genetic variations called single‐nucleotide polymorphisms (SNPs) to predict traits, using summary statistics from existing genome‐wide association studies (GWAS) to identify predictive variants. Whereas these GWAS may have sample sizes in the hundreds of thousands if not millions, polygenic scores themselves have fairly modest statistical power requirements, being potentially predictive with as few as 100 participants [27]. They are applicable to general population samples without close family members or uncommon familial relationships. Finally, the SNP‐based genotyping they require is relatively cheap, making their addition to existing research cohorts reasonably accessible. These advantages have driven a proliferation of studies attempting to use polygenic scores as controls for genetic confounding across a variety of phenotypes, with results largely supporting the independence of genetic and environmental predictors [28, 29, 30, 31, 32, 33, 34].

However, polygenic scores carry a number of limitations that may impact their suitability as a control for genetic confounding. All SNP‐based methods are limited to measuring SNP heritability, the genetic variation which can be explained by the additive effects of common SNPs. This omits variation from rare genetic variants and non‐additive genetic effects such as dominance and epistasis, resulting in estimates for total SNP heritability typically being around half the total heritability estimated by twin and family studies [35, 36]. Even within this limitation, rather than explaining the total SNP heritability of a trait, the predictive power of polygenic scores is dependent upon the size of the GWAS from which they were derived [37, 38]. While polygenic scores derived from larger GWAS like those of educational attainment are capable of predicting between 12% and 16% of the variance in the trait [39], quite near the predicted SNP heritability, most traits are limited to weaker polygenic scores capable of predicting only a small percentage of the trait. Consequently, polygenic scores may only be incomplete genetic controls, with residual correlations after using them as such still potentially confounded by both additive and non‐additive genetic effects [9]. In light of these limitations, previous studies suggesting that the independence of polygenic and environmental prediction indicates a lack of genetic confounding are likely premature [40].

Furthermore, polygenic scores are themselves potentially confounded and inflated by indirect genetic effects [41]. One important example of this is genetic nurture, where environments causal to an outcome of interest occur as a result of genes present in the parents or other close genetic relatives [42]. Such genes would be passed down to offspring and thereby associated with the outcome of interest in a way detectable by GWAS, giving the false impression of genetic rather than environmental causality in the offspring. Indeed, it is estimated that approximately half the effect of the aforementioned polygenic score for educational attainment can be explained by genetic nurture rather than direct genetic effects [39]. Population structure is also a concern, occurring when genetically distinguishable subpopulations are associated with different environments (often as a consequence of their inhabiting separate geographic regions), meaning environment now correlates with genetic differences. And while population structure is generally controlled for with the inclusion of ancestry principal components, an emerging body of research suggests that residual stratification may remain [43, 44, 45, 46]. However, methods for detecting and adjusting for indirect genetic effects typically require genotyped parents or siblings, undercutting the advantage of applying polygenic scores (PGS) in more general samples [47, 48].

Overall, it is unclear if the advantages of polygenic scores as an accessible form of genetic control outweigh their disadvantages in terms of the inferences researchers can reliably make from their results. To our knowledge, only one study to date has attempted to use a polygenic score of EF as a potential control for genetic confounding, examining its relationship with family income in the ABCD cohort [32]. This study failed to find any overlap in the genetic and environmental predictors. However, as there are no comparable studies examining genetic confounding between EF and family income, it is difficult to determine if these results truly evidence a lack of genetic confounding or simply the limitations of PGS in detecting genetic confounding. Furthermore, while the study examined indirect genetic effects via a within‐family model, between‐family prediction of EF by the PGS was also non‐significant, likely pointing to limitations of sample power. While they did find evidence of indirect genetic effects for the EF PGS on another construct, a learning and memory factor, it is unclear from these findings if indirect genetic effects are present for polygenic prediction of EF itself.

In this study, we attempt to better clarify the value of EF polygenic scores as a genetic control by evaluating their ability to control for genetic confounding in a childhood sample. We focus on maternal education, neighborhood‐level socio‐economic status, prenatal smoking, and single parenthood as environmental factors. Importantly, the measures used for these environmental variables have been previously examined for genetic confounding in another UK‐based cohort [25], meaning that agreement or disagreement between these results will speak more clearly to the strengths and limitations of EF PGS. Furthermore, our study is the first to our knowledge to use a multiethnic sample for polygenic prediction in EF, examining both European and South Asian ancestry participants. First, we examine the predictive power of the EF GWAS in children, both for EF itself and for measures of academic achievement and social development, which being traits influenced by EF should also theoretically be predictable from EF genetics. We then evaluate the value of EF polygenic scores as a control for genetic confounding by examining the effect of environmental predictors both with and without the PGS included as a control. Finally, we examine if there is evidence of indirect genetic effects confounding the EF PGS by including the mother's PGS as a control.

## 2 Methods

### 2.1 Cohort

Born in Bradford is a longitudinal birth cohort, which enrolled pregnant women living in Bradford, England between 2007 and 2011 [49]. A total of 12,453 women were enrolled at baseline, covering 13,776 pregnancies (women could re‐enrol with additional pregnancies), with 11,396 of the resulting children subsequently enrolled in the cohort. The cohort is notable for its oversampling of participants of British Pakistani origin (~45% of the cohort), who make up a significant proportion of Bradford's population. Data used for this study was collected at baseline during and immediately following pregnancy [49], during the Growing Up in Bradford follow up conducted at age 7–11 [50], and via education record data linked to the cohort through the cooperation of the Bradford Metropolitan District Council. Data access was granted upon application with the Born in Bradford study team.

### 2.2 EF Measures

EF measures were collected as part of the primary school years follow up, which occurred at a mean age of 8.45 ± 0.67 years. Full materials and procedures for all Born in Bradford cognitive tasks are described in detail in a dedicated paper [51], and so descriptions here will be brief. Three tasks—Flanker, Backwards digit recall, and Corsi block tapping—were used to measure EF.

The Flanker task, an adaptation of the Eriksen Flanker Task [52], is a measure of inhibition. The task requires children to quickly identify the direction of a central arrow, which is flanked either by arrows pointing in the same direction as the central arrow (congruous trials) or in the opposite direction (incongruous trials). Mean reaction time for correctly answered congruent trials was subtracted from mean reaction time for correctly answered incongruent trials to obtain the final outcome measure. This is in line with standard usage of the task [52, 53, 54] and is more appropriate than accuracy alone due to the task's substantial ceiling effect for accuracy, with over 48% of BiB participants correctly answering all incongruent trials.

Backwards Digit Recall [55] is a measure of verbal working memory (WM). The task requires children to listen to a sequence of numbers and repeat it back in reverse order, with sequence length increasing in later trials. Sequence length began at two and increased to a maximum of five, with each participant completing four trials at each sequence length. Mean proportion correct across all trials was used as the outcome measure.

The Corsi block tapping task [56] was used as a measure of spatial WM. The task requires children to attend to a screen, where a sequence of blocks is shown lighting up. Children are then asked to repeat this sequence once it is completed by tapping the blocks in order, with sequence length increasing between trials. Sequence length began at three and increased to a maximum of six, with each participant completing four trials at each sequence length. Mean proportion correct across all trials was used as the outcome measure.

The sample was reduced to those with both genetic and EF data and which could be assigned to the European and South Asian subsamples, on the basis of genetic ancestry data supplied by Born in Bradford (see below). Outliers for each test, defined as samples that fell outside the interquartile range by a factor of more than 1.5, were identified and removed. This resulted in an *n* of 843 for the European subsample and 1834 for the South Asian subsample. Distributions were plotted for each task and visually inspected to ensure that all outcome measures were normally distributed and showed no floor or ceiling effects.

Because measuring EF as a latent variable has been recommended to control for task impurity (non EF‐demands present in all EF tasks) [57], we used confirmatory factor analysis (CFA) to combine these three tasks into a latent factor throughout the analysis. Task correlations, shown in Table 1 for the full sample, European subsample, and South Asian subsample, were weak but highly significant, in line with previous EF research [58, 59].

**TABLE 1:** Task correlations for the EF task, in the full sample and subsamples.

	Correlation	CI lower	CI upper	*p*
Full sample				
Flanker—BDR	0.1084251	0.07083172 0	0.14571089	1.867e‐08
Flanker—Corsi	0.129855	0.09242537	0.16691818	1.54e‐11
BDR—Corsi	0.4169816	0.3851820	0.4477921	< 2.2e‐16
European subsample				
Flanker—BDR	0.1273143	0.06031047	0.19317593	0.0002105
Flanker—Corsi	0.1726244	0.1063417	0.2373798	4.594e‐07
BDR—Corsi	0.4552919	0.4000687	0.5072211	< 2.2e‐16
South Asian subsample				
Flanker—BDR	0.09917633	0.05364782	0.14429336	2.092e‐05
Flanker—Corsi	0.11119	0.06575263	0.15616729	1.812e‐06
BDR—Corsi	0.4010743	0.3619469	0.4387911	< 2.2e‐16

**FIGURE 1:**
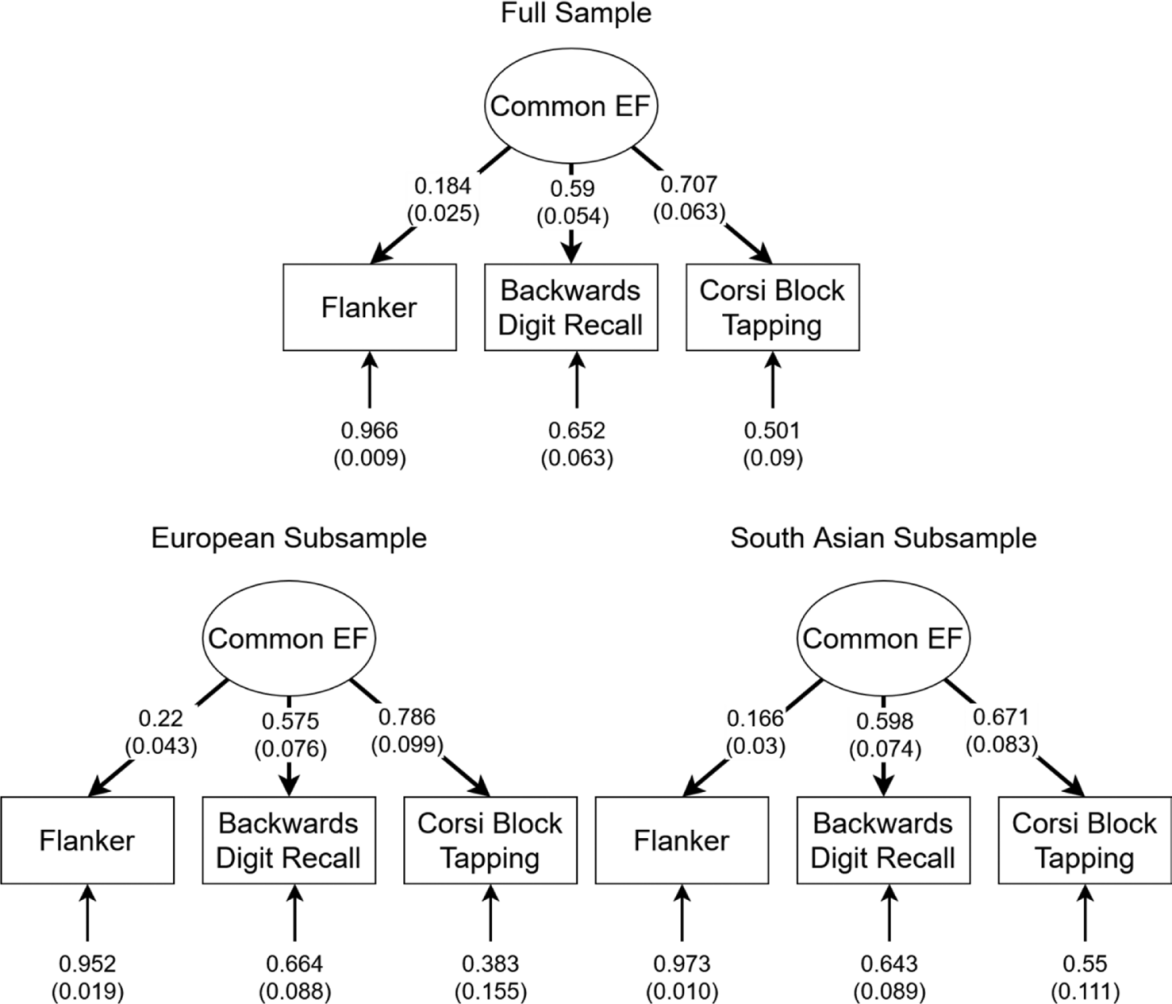
Standardized estimate for the confirmatory factor analysis of our EF measures. Standard errors in parentheses. All paths were significant at *p* < 0.001.

Analysis was performed using the lavaan package in R [60]. As the CFA used only three tasks, the result was a fully saturated model. Figure 1 displays loadings for the full and subsamples. The latent factor was dominated by the two WM tasks, possibly indicating that the latent factor reflects WM (which would include both common EF and WM‐specific variance) rather than pure common EF.

In order to determine if the EF model was invariant between the two subsamples, we used the group option in lavaan to fit a configural invariance model where the same factor structure was applied to both the European and South Asian subsamples separately. A weak invariance model constrained factor loadings to be equal between the two subsamples, and a strong invariance model constrained factor loading and intercepts to be equal between subsamples. Table 2 presents the results and comparisons between the models. None of these models were significantly different from one another, indicating that the EF model did not differ significantly between the two subsamples.

**TABLE 2:** Invariance testing for the EF model between subsamples.

	df	AIC	BIC	*χ* ^2^	Δ*χ* ^2^	RMSEA	Δdf	*p*
Configural invariance	0	−4570.8	−4464.7	0.0000				
Weak invariance	2	−4573.1	−4478.8	1.6832	1.6831	0.0000	2	0.431
Strong invariance	4	−4574.7	−4492.2	4.0205	2.3373	0.0112	2	0.311

*Note:* *p* values between the model and the one above it.

Having confirmed the suitability of this model, for subsequent regressions and structural equation models the EF factor model was fit in lavaan simultaneously with the rest of the analysis, in order to avoid indeterminacy from using a factor score. However, factor scores for the full sample were generated using the lavPredict function in lavaan for the purpose of descriptive statistics.

### 2.3 Other Socio‐Emotional and Cognitive Measures

Strengths and difficulties questionnaire (SDQ) is an informant‐report measure of psychosocial problems, with subscales for emotional symptoms, conduct problems, hyperactivity, peer relationships, and prosocial behavior [61]. The scale was completed by teachers as a part of the Born in Bradford primary school years follow‐up, mean age 8.67 (±0.55) years [50]. The total difficulty score, calculated as the sum of all subscales, was used as the outcome measure.

Early Years Foundation Stage Profile (EYFSP) is a teacher‐report measure of academic and socioemotional development. It was administered by the school at a mean age of 5.2 (±0.29) years and provided to the Born in Bradford cohort as part of linked education record data. Communication, language and literacy; problem solving, reasoning and numeracy; personal, social and emotional development; physical development; and knowledge of the world. The overall total score, obtained by summing each subsection, was used as the outcome measure. Because a different version of the test was used in the 2011/2012 academic year and 2012/2013 academic year, we standardized both waves to have a mean of 0 and standard deviation of 1 before combining the waves.

Key Stage 1 (KS1) is a standardized test used to measure educational achievement. The test was administered by the school at a mean age of 7.21 (±0.30) years and, like the EYFSP, provided to the Born in Bradford cohort as part of linked education record data. It includes subsections for maths, science, reading, and writing ability. We used the composite score, obtained by summing the results of each subsection, as the outcome measure. Also, like the EYFSP, a different version of the test was used in the 2014/2015 academic year and 2015/2016 academic year. We standardized both waves to have a mean of 0 and a standard deviation of 1 before combining the waves.

### 2.4 Environmental Variables

All environmental variables were collected during pregnancy at the time of initial Born in Bradford enrollment, via a self‐report questionnaire completed by the mother [49]. Maternal education was measured using national vocational qualification levels (NVQ), ranging from 1 (low GCSE) to 5 (graduate degree). While the Born in Bradford cohort had already equivalized educational qualifications into NVQ levels, in doing so they merged NVQ levels 4 and 5 into a single category covering all university graduates. We used the non‐equivalized education data to separate participants with graduate degrees into NVQ level 5.

Socioeconomic status was measured via the index of multiple deprivation, a neighborhood‐level measure of deprivation. The index is a weighted composite of scores across seven areas: income, employment, education, health, crime, barriers to housing and services, and living environment. Scores were assigned based on postcode of residence at baseline (note that in the UK, postcodes are fairly specific, often covering only a single street). Total deprivation score was used, with higher scores corresponding to more deprivation.

Prenatal smoking was operationalized as two variables, any smoking during pregnancy and smoking severity during pregnancy. Any smoking was a binary measure taken from the “Mother smoked during pregnancy” derived score calculated by the cohort. Smoking severity was calculated using the responses to the items “Number of cigarettes smoked per day in 3 months before pregnancy” and “Number of cigarettes smoked per day since 4th month of pregnancy”, and translated to the following 7‐point scale, taken from Knopik et al. [62]:

- 1 = did not smoke during pregnancy
- 2 = smoked during first trimester only, 1–10 cigarettes/day
- 3 = smoked during first trimester only, 11–19 cigarettes/day
- 4 = smoked during first trimester only, 20+ cigarettes/day
- 5 = smoked beyond first trimester, 1–10 cigarettes/day (max of all three trimesters)
- 6 = smoked beyond first trimester, 11–19 cigarettes/day (max of all three trimesters)
- 7 = smoked beyond first trimester, 20+ cigarettes/day (max of all three trimesters)

Single parent status was a binary measure, taken from the cohabitation status item. A small portion of the cohort (0.3%) were recorded as living with a partner other than the baby's father. These were merged with the participants living with the baby's father into a single living with a partner category.

### 2.5 Genotyping and Quality Control

Genetic data was collected at baseline via umbilical cord blood samples for offspring and blood samples from mothers. Genotyping was performed on multiple arrays: versions 12v1.0, 12v1.1, and 24v1.0 of the Illumina HumanCoreExome and version 24v2.0 of the Infinium Global Screening Array. Principal Component Analysis and reported ancestry were used to split the sample into 7580 individuals of White European and 8666 individuals of South Asian ancestry. Note that the South Asian subsample is composed primarily of participants of Pakistani origin, plus a small number of participants of Indian origin who cluster with them genetically. While it is accordingly termed South Asian rather than Pakistani, other South Asian populations present in the cohort, such as those of Bangladeshi origin, do not cluster with this sample, and so this subsample should not be interpreted as pan‐South Asian. While the vast majority of SNPs were shared between the three HumanCoreExome versions, there was minimal overlap with Global Screening Array chips. This necessitated that the samples be further separated by chip manufacturer, resulting in four subgroups (two per ancestry × two per chip manufacturer) for QC and imputation. Standard GWAS QC was performed using PLINK, removing SNPs missing in more than 5% of participants, Hardy–Weinberg equilibrium exact test *p* value below 10^−6^, or with a minor allele frequency below 1%. In the final sample, a total of 892 participants of European ancestry (452 males) had both genotyping and EF phenotypical data, of which 707 (365 males) also had genotyped mothers. A total of 2040 participants of South Asian ancestry (1021 males) had both genotyping and EF phenotypical data, of which 1471 (746 males) also had genotyped mothers.

### 2.6 Polygenic Scores

We used summary statistics from Perry et al. [63] to generate the polygenic scores. This study used genomicSEM to run a latent‐factor GWAS on multiple tests of EF, generating summary statistics for a common EF and a WM factor. As this was a bifactor formulation, the WM factor was orthogonal to EF and would contain no common EF variance, in contrast to the combination of EF and WM variance our latent factor potentially reflected. Polygenic scores were calculated for both common EF and WM using PRSice‐2 [64] following the tutorial released by the method's authors [65]. As genomicSEM is limited to single ancestry cohorts, Perry et al.'s latent factor GWAS included only European participants. Consequentially, polygenic scores derived from it would be expected to be less predictive in non‐European samples. To address this, we split our sample into European and South Asian subsamples and built polygenic scores separately for each, running analyses separately. Best‐fit PGS were obtained in the European subsample at *p* value thresholds of 0.423 and 0.00055005 for common EF and WM summary statistics, respectively, while best fit PGS were obtained in the South Asian subsample at *p* value thresholds of 1 (no thresholding) for both summary statistics.

### 2.7 Statistical Analysis

In both of our subsamples, separate regression analyses were run for each of the environmental measures and both polygenic scores to test their predictive power for EF, in order to determine what genetic and environmental influences were detectable in this sample using the available measures. Age, sex, and the first 10 ancestry principal components were used as covariates. We then tested for genetic confounding for those environmental measures which were found to be predictive, using the polygenic scores found to be predictive in the same subsample. While the common approach to test for genetic confounding using PGS is to simply include them in regressions as controls [28, 30], more complex structural equation modeling is sometimes used to simultaneously estimate both the environmental and PGS effects [29, 31]. Given that there may be a true environmental effect in addition to genetic confounding, and that PGS may function as only partial genetic controls, we favored the latter approach, which would allow for easier interpretation of the extent of confounding. In order to do this, we used a modification of mediation analysis which reversed the direction of the relationship between the independent variable and third variable (mediator/confounder), leveraging the statistical equivalence between mediation and confounding [66]. The resulting confounding model was fit for each outcome measure where both a polygenic score and an environmental variable were found to be predictive, to determine if the PGS confounded the relationship between the environmental variable and EF. All analyses were performed using the lavaan package in R [60].

We also tested the predictive power of cognitive measures theoretically downstream of EF in development, these being the SDQ, EYFSP, and KS1. As the SDQ was a teacher‐report measure, we included teacher age and sex in addition to our other covariates in those analyses (such covariates would have ideally been included for the EYFSP as well, but they were not collected by the cohort). To test the PGS for indirect genetic effects for both EF and the other cognitive measures where it was found to be predictive, we included the maternal PGS as a control, an approach that has been used in the Born in Bradford cohort previously [67]. We again used confounding models for this analysis, in order to simultaneously estimate both the direct and indirect effects of the PGS.

## 3 Results

Table 3 presents descriptive statistics for all study variables, including sample size and mean values for both subsamples, as well as *t* test *p* values indicating if the two subsamples differed on the variable. The European subsample had worse EF, more prenatal smoking, better IMD, more single parents, and a better early years' foundational stage profile compared to the South Asian subsample. The subsamples were not significantly different in maternal education, key stage 1, and strength and difficulties questionnaire.

**TABLE 3:** Means and *t* test p values for descriptive statistics, European and South Asian subsamples.

Measure	*n* European	Mean European (SD)	*n* South Asian	Mean South Asian (SD)	*p*
Executive function	843	−0.006 (0.032)	1843	0.001 (0.034)	**< 0.01**
Maternal education	679	2.415 (1.142)	1479	2.356 (1.176)	0.2645
Smoking any	755	0.325 (0.468)	1547	0.03 (0.172)	**< 0.001**
Smoking severity	756	2.696 (2.613)	1552	1.151 (0.919)	**< 0.001**
IMD	756	37.278 (18.937)	1552	47.548 (14.052)	**< 0.001**
Single parent status	753	1.259 (0.438)	1547	1.059 (0.237)	**< 0.001**
Psychosocial problems (SDQ)	124	5.798 (5.838)	296	6.848 (6.099)	0.099
Academic and socioemotional development (EYFSP)	836	0.232 (0.898)	1814	0.036 (0.950)	**< 0.001**
Academic achievement (KS1)	839	0.146 (0.832)	1819	0.112 (0.856)	0.330

*Note:* Bolded values are significant at *p* < 0.05. Abbreviations: EYFSP, Early Years Foundation Stage Profile, IMD, index of multiple deprivation; SDQ, strengths and difficulties questionnaire.

In order to test the individual predictive power of our measures before any potential confounding is accounted for, we performed linear regressions of the polygenic scores and each of the environmental predictors on EF separately. Table 4 presents results in the European subsample. Neither polygenic score was predictive of EF in this subsample, rendering them unsuitable for confounding analysis. Maternal education, any smoking, and IMD were significant predictors of EF, while smoking severity and single parent status fell just short of significance.

**TABLE 4:** Linear regression results for the PGS and environmental predictors of executive function in the European subsample.

Predictor	*n*	Unstandardized estimate	Standardized estimate	*z* score	*p*
EF polygenic score	843	0.003 (0.002)	0.074 (0.043)	1.693	0.09
WM polygenic score	843	−0.001 (0.002)	−0.029 (0.043)	−0.690	0.49
Maternal education	679	0.007 (0.003)	0.156 (0.047)	3.296	**< 0.001**
Smoking any	755	−0.009 (0.005)	−0.082 0.044	−1.850	0.064
Smoking severity	756	−0.002 (0.001)	−0.092 (0.044)	−2.085	**< 0.05**
IMD	756	−0.000 (0.000)	−0.093 (0.045)	−2.07	**< 0.05**
Single parenthood	753	−0.011 (0.006)	−0.084 0.045	−1.854	0.064

*Note:* Bolded values are significant at *p* < 0.05. Standard errors in parentheses.

Table 5 presents results for the same series of linear regressions in the South Asian subsample. The EF polygenic score was a stronger predictor in this subsample than in the European subsample. The WM polygenic score also significantly predicted EF, although this was fully attenuated when the EF polygenic score was also included in the regression (*z* = 1.505, *p* = 0.132). Maternal education was the only significant environmental predictor of EF in this subsample.

**TABLE 5:** Linear regression results for the PGS and environmental predictors of executive function in the South Asian subsample.

Predictor	*n*	Unstandardized estimate	Standardized estimate	*z* score	*p*
EF polygenic score	1843	0.007 (0.002)	0.197 (0.033)	5.937	**< 0.001**
WM polygenic score	1843	0.004 (0.002)	0.105 (0.034)	3.092	**< 0.01**
Maternal education	1479	0.005 (0.002)	0.179 (0.034)	5.256	**< 0.001**
Smoking any	1547	0.002 (0.007)	0.011 (0.033)	0.339	0.735
Smoking severity	1552	0.002 (0.001)	0.038 (0.033)	1.157	0.247
IMD	1552	0.000 (0.000)	0.012 (0.033)	0.350	0.727
Single parenthood	1547	−0.003 (0.005)	−0.016 (0.033)	−0.495	0.621

*Note:* Bolded values are significant at *p* < 0.05. Standard errors are in parentheses.

**FIGURE 2:**
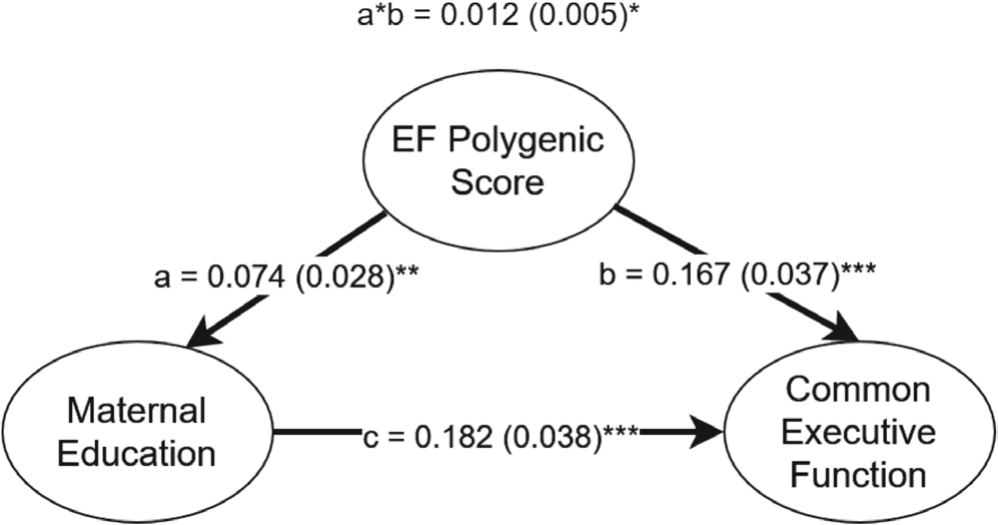
Confounding model for EF PGS and maternal education in the South Asian subsample. a*b represents the confounding pathway. *p < 0.05, **p < 0.01, ***p < 0.001.

We then attempt to control for genetic confounding with the EF PGS. Because the EF PGS was only predictive in the South Asian subsample, and maternal education was the only environmental predictor of EF in this group, only maternal education could be assessed for potential genetic confounding. Figure 2 presents the results of this confounding model. The confounding pathway accounted for a small but statistically significant (*z* = 2.358, *p* < 0.05) fraction of the variance, indicating that genetic confounding is present in this sample. However, maternal education remained a highly significant predictor of EF (*z* = 4.781, *p* < 0.001), with only a modest reduction in effect size compared to the model without the PGS as a control.

We then go on to examine the predictive power of the PGS for non‐EF cognitive outcomes. Tables 6 and 7 present the results of separate linear regressions for the EF and WM polygenic scores on academic achievement as assessed by the Key Stage 1, academic and socioemotional development as assessed by the EYFSP, and psychosocial problems as assessed by the SDQ. The PGS was predictive of academic achievement (KS1) in both samples, while it was only predictive of academic and socioemotional development (EYFSP) in the South Asian subsample. The EF polygenic scores were not predictive of psychosocial problems (SDQ) in either sample, likely due to the low sample size of this measure. The WM polygenic score was only predictive in the South Asian subsample for academic and socioemotional development (EYFSP) and academic achievement (KS1). However, as with EF, this attenuated when the EF polygenic score was also included (*z* = 5.741, *p* = 0.092 and *z* = −0.425, *p* = 0.671, respectively).

**TABLE 6:** Predictive power of the EF PGS on other socio‐emotional and cognitive outcomes in the European subsample.

Cognitive measure	*n*	Unstandardized estimate	Standardized estimate	*z* score	*p*
EF PGS					
Psychosocial problems (SDQ)	124	0.69 (0.527)	0.124 (0.094)	1.321	0.186
Academic and socioemotional development (EYFSP)	836	0.021 (0.032)	0.023 (0.035)	0.653	0.514
Academic schievement (KS1)	839	0.064 (0.030)	0.077 (0.037)	2.116	**< 0.05**
WM PGS					
Psychosocial problems (SDQ)	124	0.311 (0.552)	0.058 (0.102)	0.563	0.573
Academic and socioemotional development (EYFSP)	836	−0.005 (0.032)	−0.005 (0.035)	−0.153	0.879
Academic achievement (KS1)	839	−0.024 (0.03)	−0.029 (0.037)	−0.800	0.424

*Note:* Bolded values are significant at *p* < 0.05. Standard errors in parentheses.

**TABLE 7:** Predictive power of the EF PGS on other socio‐emotional and cognitive outcomes in the South Asian subsample.

Cognitive measure	*n*	Unstandardized estimate	Standardized estimate	*z* score	*p*
EF PGS					
Psychosocial problems (SDQ)	296	−0.464 (0.391)	−0.078 (0.066)	−1.193	0.233
Academic and socioemotional development (EYFSP)	1814	0.120 (0.023)	0.125 (0.023)	5.349	**< 0.001**
Academic achievement (KS1)	1819	0.111 (0.021)	0.13 (0.024)	5.349	**< 0.001**
WM PGS					
Psychosocial problems (SDQ)	296	−0.374 (0.382)	−0.064 (0.066)	−0.980	0.327
Academic and socioemotional development (EYFSP)	1814	0.054 (0.023)	0.056 (0.024)	2.352	**< 0.05**
Academic achievement (KS1)	1819	0.057 (0.021)	0.066 (0.025)	2.687	**< 0.01**

*Note:* Bolded values are significant at *p* < 0.05. Standard errors in parentheses.

### 3.1 Indirect Genetic Effects

**FIGURE 3:**
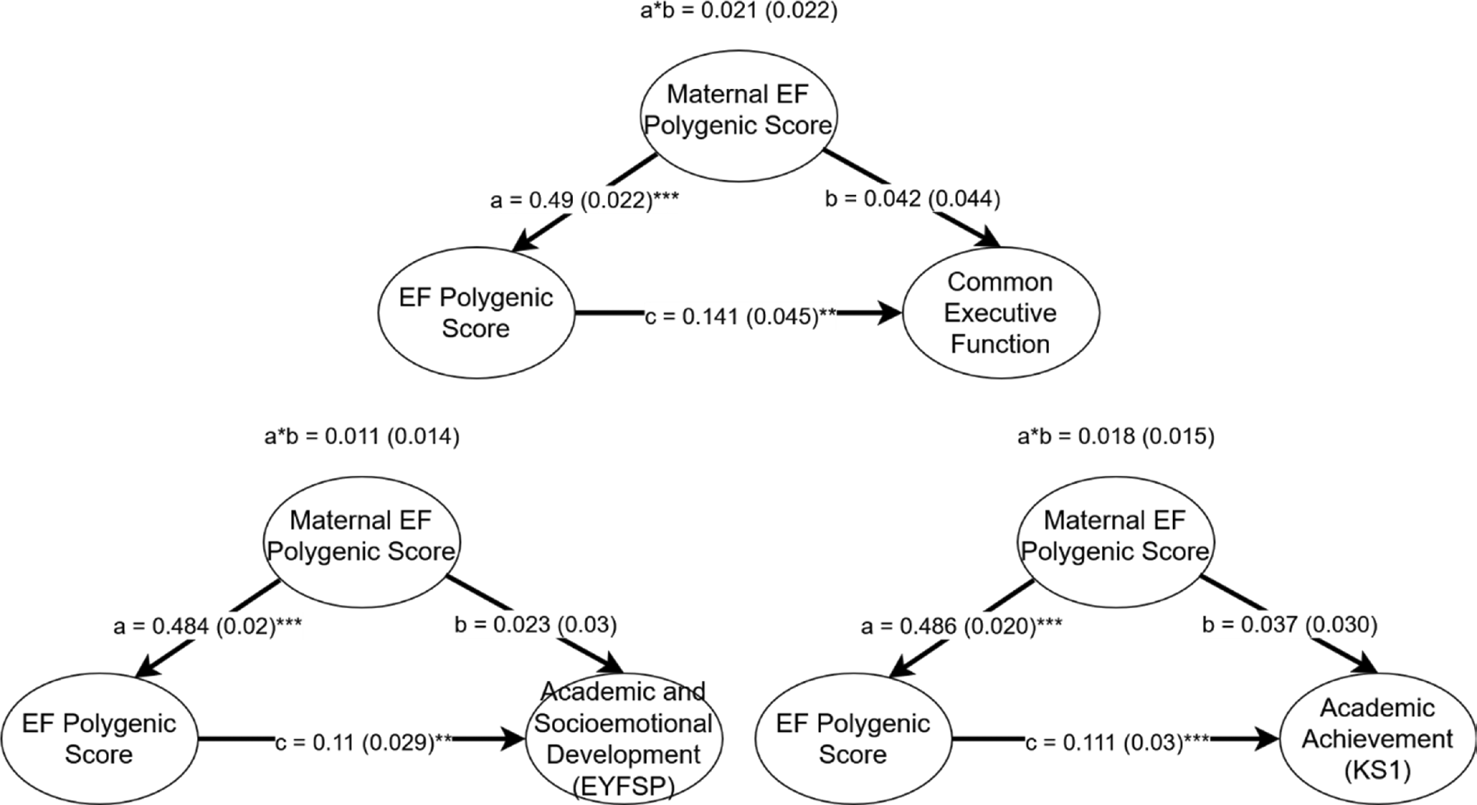
Confounding model for the effect of maternal PGS on offspring PGS prediction in the South Asian sample. a*b represents the confounding pathway. *p < 0.05, **p < 0.01, ***p < 0.001.

Indirect genetic effects could not be tested in the European subsample, as the loss in statistical power caused by restricting the sample to those with the maternal PGS was sufficient to cause the effect of EF PGS on academic achievement (KS1) to fall below statistical significance before including maternal PGS as a control. In order to first demonstrate their predictive power and thus suitability for confounding analysis, we first regressed the maternal EF PGS on EF, Academic and Socioemotional Development (EYFSP), and Academic Achievement (KS1) in the South Asian subsample. We also performed separate regressions using the offspring PGS after reducing the sample to participants with maternal PGS as well. Table 8 presents the results. For all outcomes for which the offspring EF PGS was previously predictive, maternal PGS was also a strong predictor, and the offspring PGS remained significant, despite the reduction in sample size. We next fit confounding models containing both the maternal and offspring PGS for each of these outcomes, in order to test for indirect genetic effects of the maternal PGS. Figure 3 presents the results. In all models, cohort member's EF PGS remained a statistically significant predictor of the outcome, while maternal EF PGS and thus the confounding pathway were not predictive, suggesting indirect genetic effects of the PGS were minimal.

**TABLE 8:** Predictive power of the maternal and offspring EF PGS in the restricted South Asian subsample.

Cognitive measure	*n*	Unstandardized estimate	Standardized estimate	*z* score	*p*
Maternal PGS					
EF	1317	0.004 (0.002)	0.140 (0.039)	3.591	**< 0.001**
Academic and socioemotional development (EYFSP)	1300	0.073 (0.025)	0.076 (0.026)	2.940	**< 0.01**
Academic achievement (KS1)	1307	0.077 (0.023)	0.091 (0.027)	3.417	**< 0.001**
Offspring PGS					
EF	1317	0.005 (0.002)	0.162 (0.039)	4.163	**< 0.001**
Academic and socioemotional development (EYFSP)	1300	0.115 (0.024)	0.12 (0.026)	4.732	**< 0.001**
Academic achievement (KS1)	1307	0.108 (0.022)	0.129 (0.026)	4.920	**< 0.001**

*Note:* Bolded values are significant at *p* < 0.05. Standard errors in parentheses.

### 3.2 Additional Analysis

**FIGURE 4:**
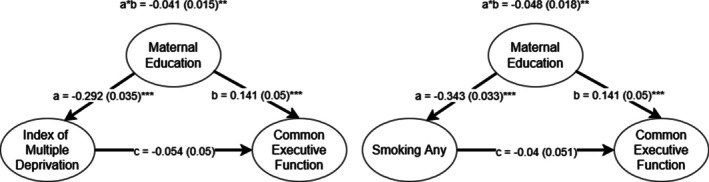
Confounding models for maternal education and other environmental predictors in the European subsample. a*b represents the confounding pathway. *p < 0.05, **p < 0.01, ***p < 0.001.

Differences between environmental predictors of EF in the European and South Asian subsamples suggested additional analyses. The South Asian subsample had better EF despite experiencing more deprivation as measured by IMD. However, IMD did not significantly predict EF in the South Asian subsamples, while maternal education, which the two subsamples did not significantly differ on, was predictive in both. We would expect some relationship between maternal education and IMD, both because it includes average neighborhood education level in its composite, and because it includes other metrics influenced by education such as income. However, 68.8% of South Asian mothers in the Born in Bradford cohort were unemployed, compared to 25.3% of European mothers [68], meaning that neighborhood‐level SES and thus IMD might have been more influenced by the paternal education and earning capacity in the South Asian subsample. Therefore, one possibility is that the predictive power of IMD in the European subsample alone is simply due to it better capturing maternal education in that subsample. Similarly, any prenatal smoking was only predictive of EF in the European subsample. While this can be plausibly explained by low variance in the South Asian subsample (which contained very few smokers), it still might suggest these variables are secondary in importance to maternal education. In order to test this, we fit confounding models to estimate the effect of IMD and any prenatal smoking on EF after controlling for confounding via their association with maternal education. Figure 4 presents the results. Consistent with our hypothesis, confounding by maternal education explained all of the variance, with IMD and prenatal smoking not significantly predicting EF once maternal education was accounted for.

## 4 Discussion

Our results provide weak evidence of genetic confounding in EF. Maternal education was the only significant environmental predictor of EF across both subsamples, which in the South Asian subsample was confounded by the EF polygenic score. These results contrast with those using a previous iteration of the EF polygenic score, for which family income and the EF polygenic score were completely independent predictors [32]. However, the confounding detected in our study was much weaker than what was shown in previous research using GCTA [25]. The direct effect of maternal education remained significant when potential confounding by the EF PGS was included in the model and, in fact, explained much more of the variance than said indirect effect. The European subsample uniquely showed an effect of IMD and any prenatal smoking on EF outcomes. However, the polygenic score was much weaker in its predictive power in this subsample, making it unsuitable for confounding analysis. As a consequence, our results are far more limited than those obtained using specialized samples for genetically sensitive designs, suggesting that the polygenic score of EF may not be well positioned to replicate the findings of these studies.

Despite these limitations, our study was well positioned to detect indirect genetic effects. In addition to predicting EF, the EF PGS was a significant predictor of both the KS1 (measuring academic achievement) and the EYFSP (measuring academic and socioemotional development) in the South Asian subsample. However, maternal EF PGS did not significantly predict any of these traits independently of offspring PGS. Previous work also returned null results for indirect effects from both maternal genetics and genetic trios [25], suggesting that indirect genetic effects on EF are either non‐existent or too small to be detected with these methods. This question was also examined by the previous study of the EF PGS; however, as both the between‐family and within‐family effect of the EF PGS on EF were non‐significant in their model, their sample was insufficiently powered to detect indirect genetic effects [32], much as our European subsample was. Given that polygenic scores are better positioned to detect small effect sizes compared to previous GCTA methods, particularly in a larger sample like our South Asian subsample, our results further constrict the potential effect size of indirect genetic effects. Additionally, consanguinity, which is common in the Born in Bradford's South Asian population [49] may inflate estimates of indirect genetic effects [69], making it particularly notable that none were detected here. Indirect genetic effects are a common problem in related cognitive phenotypes such as educational attainment [47, 48]. However, according to twin studies, EF diverges from these traits in its lack of shared environmental influence. Because genetic nurture is thought to operate along environmental pathways which would be expected to be shared in twins, one might predict that EF lacks the mechanism that allows genetic nurture to occur, which is consistent with our results. These results therefore support the claim that shared environment is not operative in EF and indicate that genes which are associated with EF are unlikely to be tapping indirect genetic effects.

Our results also provide insight into the relative importance of certain environmental predictors of EF. While IMD and any prenatal smoking were both significantly associated with EF in the European subsample, confounding models showed that the predictive power of these variables on EF could be entirely explained by maternal education. Many studies use maternal education as a proxy for socio‐economic status, despite the latter typically being conceptualized as a multidimensional construct [2, 70, 71]. IMD arguably does a better job of capturing this multidimensionality despite being a neighborhood‐level measure, as it is a composite including measures of income, employment, crime, and health in addition to education. However, our analysis found this contributed no additional information beyond maternal education itself. Previous research has recommended that the components of SES be studied individually rather than subsumed under the larger construct, as they can produce differing effects and better illuminate the relevant mechanisms [70, 72, 73]. Our analysis likewise suggests that a focus on maternal education over broader conceptions of SES is likely to tap pathways more directly relevant to EF development. And while prenatal smoking is more frequently conceptualized as a biochemical influence on development, it also correlates with a number of sociodemographic characteristics, including the mother's educational attainment [74], which may confound its prediction of EF. Consistent with this, we also found that the predictive power of any prenatal smoking could be entirely explained by maternal education. There were therefore no environmental predictors that explained variation in EF beyond that of maternal education. However, maternal education still explained < 3% of the variance in EF outcomes. Furthermore, the mechanism underlying this association is unclear, with maternal education possibly being a simple index of more proximate environments such as family behaviors [75]. Thus, while our findings support maternal education as the most important of the studied variables for EF outcomes and support investigation into potential mediators, they also suggest that researchers should consider a wider range of variables less closely linked to maternal education in order to better understand influences on EF development.

A major limitation of this study was the reduced predictive power of the EF PGS in the European subsample compared to the South Asian subsample. Previous work has suggested that polygenic scores derived from European samples are only ~60% as predictive when used in a South Asian sample [76], making it surprising that ours would perform better in the latter. While it may be tempting to attribute this to the larger sample size of the South Asian subsample, effect sizes in the European subsample were smaller, and sensitivity analysis showed that the South Asian subsample could be restricted to a sample size as low as 350 without the EF PGS losing statistical significance. The reason for this discrepancy is thus unclear. Given that the European subsample had worse EF performance than the South Asian subsample, one possibility is that some environmental effect is impacting task performance in the European subsample and therefore reducing the reliability of genetic prediction. Testing environments may have been less ideal or more heterogeneous for the European subsample. For instance, as 86 different schools participated in EF data collection, and the South Asian population in Bradford is more tightly geographically clustered than the European population [77], it is likely that the European participants were spread across a larger number of schools and thus potentially experienced a more heterogeneous testing environment. There may also have been more variation in familiarity with the computerized testing materials. While our use of a latent factor was intended to minimize the effects of measurement error such as these, the smaller EF test battery would have been limited in its ability to do so, particularly if the same sources of measurement error were present across tasks. It is also possible that, as potential nesting within schools was not controlled for statistically, results in one or both subsamples were upwardly biased due to non‐independence. However, upward bias in PGS prediction due to nesting within schools would require school placement to be influenced by genetic nurture, which our results do not support as being active in EF. This bias would thus be more likely to influence estimates of environmental influences on EF, or possibly EF influences on downstream outcomes. Finally, previous research has suggested that PGS may be more or less predictive across certain environments [67]. This research did focus on variation in SES, which given our other findings seems unlikely to explain the differential prediction observed here. However, it is possible there are other environments that also produce this effect. As limitations owing to the homogeneity of typical twin samples are a possible explanation for reports of low shared environmental influences, it is important that studies consider how increasing the range of environmental exposure may alter results.

Finally, it is important to consider how the limitations of polygenic prediction in general may have impacted our results. Rather than reflecting full‐scale heritability, polygenic scores are limited to heritability due to common SNPs. And even within that, rather than estimating full SNP heritability, the predictive power of polygenic scores is dependent upon the GWAS from which they are derived, with large GWAS of the same trait producing more predictive scores [37, 38]. The absolute predictive power of the EF polygenic score is thus likely more reflective of the size of current EF GWAS than the trait's true SNP heritability. Furthermore, the GWAS from which our polygenic score was derived relied on an imperfect measure of EF. The first large‐scale GWAS of EF, conducted in the UK Biobank, used a latent factor that included typical EF tasks but also other cognitive tasks not typically used to measure EF but with hypothesized EF demands [78]. This was further refined by Perry et al. [63] who used genomicSEM to include GWAS of other EF tasks, as well as GWAS of principal components with loadings from EF tasks. While the inclusion of these nonstandard measures improved statistical power, they may have also moved the construct away from pure EF, potentially leading it to capture more general cognitive ability, processing speed, or some other related construct. Given that our EF latent factor was also an imperfect measure, being composed of only three tasks (with only the backwards digit task shared with the GWAS), dominated by WM tasks, and lacking a shifting task, the two constructs may have been less unified than would have been ideal. Perry et al.'s [63] GWAS also included a WM‐specific factor orthogonal to common EF, which was a weak predictor of our EF factor in the South Asian subsample only. This may reflect a larger portion of EF to WM‐specific variance in our EF factor, or simply the fact that the GWAS of the WM‐specific factor had reduced statistical power compared to that of common EF, being constructed of fewer terms with weaker loadings and thus having a lower effective sample size.

## 5 Conclusion

Polygenic scores produce divergent results from other genetically‐informed methods when used to study genetic confounding in EF. More research is necessary to determine why, but in the meantime, caution must be used when treating the EF polygenic score as a genetic control. Our results support only maternal education as an environmental predictor of EF, with the association of neighborhood‐level SES and prenatal smoking with EF being entirely explainable via their association with maternal education. However, while our results do support there being some genetic confounding in the relationship between maternal education and EF, this explained only a small fraction of the effect. Finally, our results further support the idea that indirect genetic effects on EF are minimal, both for EF itself and for other cognitive outcomes theoretically downstream of EF. They thus suggest that molecular genetic studies of EF are unlikely to be significantly influenced by genetic nurture.

## Conflicts of Interest

The authors declare no conflicts of interest.

## Supporting information


**Data S1.** Supporting information.

## Data Availability

The data that support the findings of this study are available from Born in Bradford. Restrictions apply to the availability of these data, which were used under license for this study. Data are available from https://borninbradford.nhs.uk/our‐data/how‐to‐access‐data/ with the permission of Born in Bradford.
